# Effects of At-Home Bleaching on Color Stability and Surface Roughness of Single-Shade, ORMOCER-Based, and Conventional Resin Composites

**DOI:** 10.3390/dj14020124

**Published:** 2026-02-22

**Authors:** Colwin Yee, Hassan Ziada, Neamat Hassan Abubakr

**Affiliations:** 1 School of Dental Medicine, University of Nevada, Las Vegas, NV 89557, USA; 2 Department of Clinical Sciences, School of Dental Medicine, University of Nevada, Las Vegas, NV 89106, USA; hassan.ziada@unlv.edu; 3 Department of Biomedical Sciences, School of Dental Medicine, University of Nevada, Las Vegas, NV 89106, USA

**Keywords:** bleaching, resin composites, color stability, surface roughness, ORMOCER, esthetic dentistry

## Abstract

**Background/Objectives**: This study evaluated the effects of at-home bleaching on color stability (ΔE) and surface roughness (Ra) of a single-shade nanohybrid composite, an ORMOCER-based composite, and a conventional nanohybrid resin composite, acknowledging that bleaching represents only one of several clinical ageing challenges. **Methods**: One hundred and five extracted, non-carious human molars received standardized Class I restorations and were randomly allocated to five groups (n = 21): an ORMOCER-based composite (Admira Fusion), a single-shade composite (Omnichroma), Omnichroma bonded with an alternative universal adhesive, and two conventional nanohybrid composites (Filtek Supreme Ultra and Harmonize). Baseline and experimental color (CIELAB, ΔE) were measured with a spectrophotometer, and surface roughness (Ra) was measured using a 3D optical profilometer. Specimens underwent five bleaching cycles using 22% carbamide peroxide, with each cycle consisting of 8 h of bleaching followed by 16 h of storage in artificial saliva at 37 °C. Measurements were taken at baseline and after each cycle. The data were analyzed using a repeated-measures ANOVA, with bleaching cycle as the within-subject factor, the effect sizes reported as partial eta-squared (ηp^2^), and the statistical significance set at α = 0.05. **Results**: All restorative materials exhibited progressive color change with repeated bleaching, and ΔE values exceeded established clinical acceptability thresholds across materials. The extent of color change varied among materials. None of the evaluated materials maintained clinically acceptable color stability following repeated bleaching cycles. The single-shade composite (Omnichroma) demonstrated the greatest magnitude of color change, particularly when bonded with Scotchbond Universal Bond. Admira Fusion and Filtek Supreme Ultra had lower ΔE values but still exceeded acceptability thresholds. Surface roughness generally decreased following bleaching, with statistically significant reductions in Ra observed for multiple materials. Admira Fusion and Omnichroma bonded with Tokuyama Universal Bond showed minimal surface alteration. **Conclusions:** All restorative materials demonstrated clinically unacceptable color changes following bleaching, indicating limited esthetic stability under bleaching conditions. ORMOCER-based composites showed comparatively greater resistance to surface roughness alterations.

## 1. Introduction

The esthetic integration of composite resin restorations with natural teeth relies on accurate shade matching, controlled layering techniques, and careful surface characterization. Conventional multi-shade composite systems employ stratified materials with different optical properties, particularly variations in translucency and opacity, to reproduce the structure of enamel and dentin [1,2]. Although these can achieve reliable esthetic results, they are technique-sensitive and often require additional chairside time.

Single-shade composite resins were developed to simplify restorative procedures by reducing shade selection complexity and relying on optical blending (the “chameleon effect”); however, their esthetic performance may be influenced by material chemistry and environmental aging factors. Visual color adaptation is governed mainly by filler morphology and the way these particles interact with incident light. Nevertheless, in specific composite systems, the restricted range of available shades may limit the ability to achieve optimal color reproduction [3]. For example, Omnichroma incorporates uniformly sized spherical filler particles with an average diameter of about 260 nm. This particle size is selected to improve light scattering in the red-to-yellow wavelength range and facilitate structural color formation. The resulting optical effect is influenced mainly by the uniformity of the filler particles and the refractive index relationship between the fillers and the resin matrix, rather than by conventional pigment-based coloration. By comparison, Admira Fusion is formulated with an ORMOCER-based resin matrix. It follows a “universal shade” concept that relies on the inherent optical behavior of its hybrid inorganic–organic network, thereby reducing the need for shade stratification or added chromatic fillers.

Although favorable immediate esthetic outcomes have been reported for single-shade composites [3], evidence regarding their long-term color stability and surface integrity remains limited, particularly under clinically relevant aging conditions. Comparative investigations assessing different single-shade systems, particularly those based on distinct material chemistries such as methacrylate-based and ORMOCER-based formulations, are scarce.

Dental bleaching is a conservative and widely accepted method for improving tooth color [4,5,6]; however, restorative materials are also routinely exposed to chromogenic beverages and tobacco smoke, which may further influence long-term color stability [7,8]. Most bleaching agents contain hydrogen peroxide or carbamide peroxide, which decomposes into hydrogen peroxide and urea, subsequently releasing reactive oxygen species [9,10,11,12]. While effective for teeth whitening, bleaching agents may alter the surface and optical properties of restorative materials; therefore, evaluating bleaching-related color stability represents only one aspect of clinical aging [13]. At-home bleaching products, typically delivered using trays or adhesive strips, are widely used and applied for periods ranging from several minutes to a few hours per day [14,15]. Although the effects of bleaching agents on conventional resin composites have been extensively studied, limited information is available regarding how at-home bleaching protocols influence the optical behavior of single-shade composite materials, and this study does not account for staining or smoke-related discoloration challenges.

Color differences in dental research are commonly quantified using the Commission Internationale de l’Éclairage (CIE) color system. The ΔE values express the perceptibility and acceptability thresholds. In vitro studies generally consider ΔE values of up to 3.7 as clinically acceptable. The in vivo thresholds are typically lower, approximately 3.3 [16,17]. The clinical acceptability in this context refers to perceptibility thresholds rather than relative material performance. Therefore, comparisons among materials reflect their relative resistance or susceptibility to bleaching-induced changes rather than their ability to maintain clinically acceptable esthetic outcomes over time.

This study tested the null hypotheses that at-home bleaching does not significantly affect the surface roughness or color of composite restorative materials and that no significant differences exist among materials or adhesive systems in their response to bleaching, acknowledging that bleaching represents only one dimension of clinical aging. The present study investigated the effects of at-home carbamide peroxide bleaching on the surface roughness (Ra) and color stability (ΔE) of several composite restorative materials and determined whether observed color changes exceeded established clinical acceptability thresholds, while recognizing the absence of staining and smoke exposure as a limitation.

## 2. Materials and Methods

### 2.1. Sample Size Calculation

**Figure 1:**
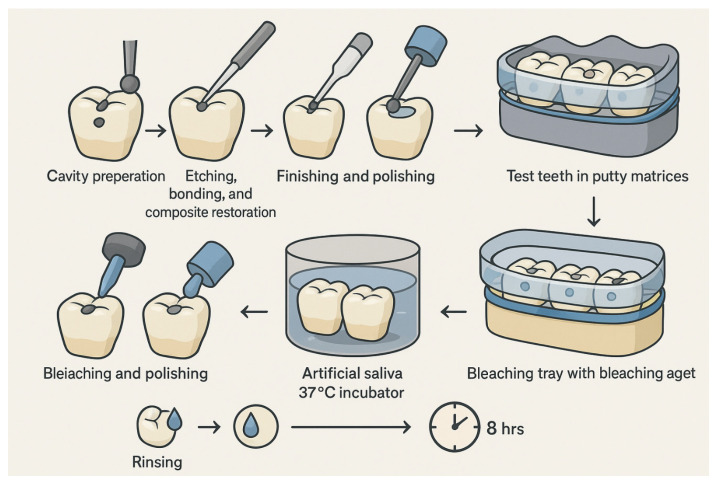
Schematic diagram of the preparation of the sample teeth.

The sample size was determined to be 100 teeth based on an alpha level of 0.05, a beta error of 0.20, and a statistical power of 80%, using an effect size reported in a previously published study on bleaching-induced color changes [16]. Of note, the study was not specifically powered to isolate adhesive-related effects. To account for the possibility of specimen loss or damage during the experimental procedures, the sample size was increased to 105 teeth. Figure 1 shows the experimental workflow.

### 2.2. Ethical Considerations

This study used anonymized, extracted, non-carious human molars removed for reasons unrelated to the present investigation. In line with institutional guidelines, ethical approval was not required for the use of extracted teeth in in vitro research, given that all patients consent to the use of their teeth for research purposes at the start of their attendance and admission to the school clinics. All experimental procedures were conducted in accordance with the ethical principles of the Declaration of Helsinki, as applicable to laboratory-based studies.

### 2.3. Sample Preparations

The teeth were cleaned and stored until ready to use. Standardized experimental Class I cavities measuring 1.0 mm in depth and 1.0 mm in diameter were prepared on the occlusal surfaces using a high-speed handpiece with water cooling; although these dimensions facilitated standardization, they are smaller than many clinical restorations and hence, may influence bleaching-related outcomes due to a higher surface-to-volume ratio. These dimensions of the preparation were specifically designed and selected for this study. A single operator carried out all cavity preparations to reduce procedural variability; however, inherent anatomical variability among extracted molars may still have influenced optical and profilometric measurements. The specimens were then randomly allocated to five groups (n = 21). Each group represents one of the restorative materials and adhesive systems employed (Table 1).

### 2.4. Restorative Procedures

The prepared cavities were etched using Scotchbond Universal Etchant (3M, Germany) for 15 s and thoroughly rinsed with water and gently air-dried. The corresponding adhesive systems were applied according to the manufacturers’ instructions for each group, recognizing that the study design was not specifically powered to independently evaluate adhesive-related effects. Composite materials were incrementally placed, sculpted to reproduce occlusal anatomy, and polymerized using a Demi Plus LED curing light (Kerr, USA). Following restorations and polymerization, they were finished with Super-Snap Singles (Shofu, Kyoto, Japan) and polished with OneGloss Mini-Point polishers (Shofu, Japan) under light, standardized pressure. These were then embedded in putty matrices, with specimens organized by restorative material.

**Figure 2:**
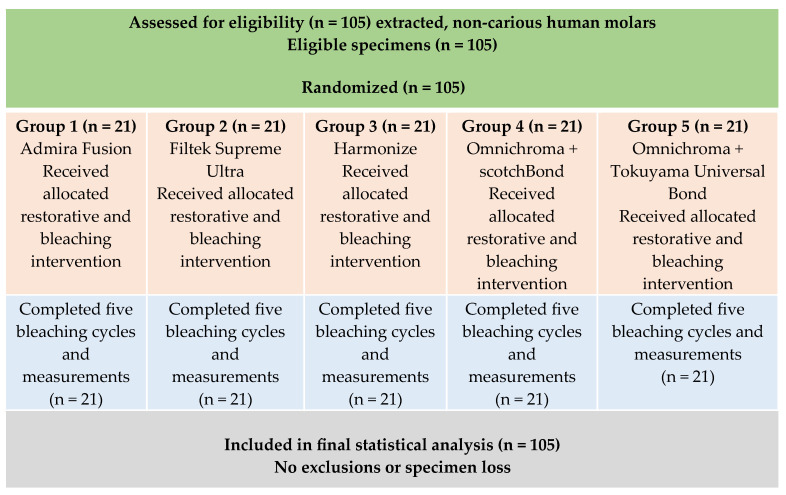
Schematic representation of the experimental workflow and bleaching protocol.

Vacuum-formed bleaching trays were fabricated over each row of specimens using thermoforming sheets. The trays were secured with rubber bands during bleaching procedures to ensure standardized contact and uniform distribution of the bleaching agent (Figure 2).

### 2.5. Bleaching Protocol

The vacuum-formed trays were designed and fabricated to provide consistent contact between the bleaching agent and the restorations. A standardized 2 mm layer of 22% carbamide peroxide gel (PolaNight, SDI Limited, Bayswater, Victoria, Australia) was applied to each restoration with the vacuum-formed tray, representing a commonly used at-home bleaching concentration.The specimens were then incubated in stabilized artificial saliva (1700-0305 Artificial Saliva, Pickering Laboratories, Mountain View, CA, USA) at 37 °C for 8 h, rinsed, assessed, and subsequently stored in artificial saliva for an additional 16 h. Each cycle represented one simulated day of at-home bleaching and was repeated for five consecutive days; however, only a single bleaching protocol was evaluated, and no non-bleached control group was included to isolate bleaching-specific effects from aging or storage influences. The artificial saliva was refreshed to simulate daily oral conditions. Figure 2 shows the experimental workflow.

### 2.6. Measurements

Baseline measurements were recorded before bleaching for both color stability and surface roughness measurements. The measurements for both parameters were as follows:

#### 2.6.1. Color Stability

Color parameters (CIELAB: L*, a*, b*; ΔE) were measured using a spectrophotometer (VITA Easyshade V, VITA, Bad Säckingen, Germany). The device was calibrated before each measurement according to the manufacturer’s instructions; however, randomization of measurement order and formal intra-observer repeatability assessment were not performed. Measurements were taken on the occlusal surface of each restoration.

#### 2.6.2. Surface Roughness Measurement

Surface roughness (Ra; average roughness) was measured using a calibrated three-dimensional non-contact optical profilometer (Keyence VR-3100, Keyence, Osaka, Japan). The profilometer provided Ra values for quantitative comparison and enabled acquisition of three-dimensional surface topography, facilitating standardized surface characterization across all specimens. For each specimen, the longest continuous section of the composite restoration was analyzed consistently after each bleaching cycle. Tilt correction was performed to minimize the effects of surface angulation or slope, ensuring accurate, reproducible, and comparable surface roughness measurements.

### 2.7. Statistical Analysis

For statistical analysis, repeated measures mixed-design ANOVA was conducted. Bleaching cycles (baseline and 5 cycles) were treated as within-subject factors, while restorative material and adhesive system were between-subject factors. This is to evaluate the effects of bleaching cycles, restorative material, and their interactions. When significant interactions or material-specific trends were identified, a separate within-material repeated-measures ANOVA was conducted to evaluate time-dependent changes within each material. Secondary analyses characterized material-specific responses and were considered exploratory, and no adjustments for multiple post hoc comparisons were applied. Data were evaluated for normality and homogeneity of variance prior to statistical testing. The effect sizes were reported as partial eta squared (ηp^2^), and statistical significance was defined as *p* < 0.05; statistical significance was interpreted separately from clinical relevance thresholds.

## 3. Results

The results are presented to describe the relative performance of restorative materials under bleaching conditions, recognizing that all tested materials exceeded established clinical acceptability thresholds for color change during the experimental period. All specimens completed the experimental protocol without damage or exclusion. The data distribution assessed, confirmed normality and homogeneity of variance, meeting the assumptions required for repeated-measures analysis. The color changes (ΔE) and surface roughness (Ra) were analyzed using a repeated-measures ANOVA with bleaching cycle as the within-subject factor and the restorative material as the between-subject factor. Statistical significance was set at *p* < 0.05, and effect sizes were reported as partial eta-squared (ηp^2^). The mixed-design repeated-measures ANOVA revealed a statistically significant main effect of bleaching cycle and material-dependent interaction trends.

### 3.1. Color Stability (ΔE)

The mean ΔE values for all restorative materials increased progressively with repeated bleaching cycles (Table 2). All materials exceeded the commonly accepted clinical acceptability threshold (ΔE = 3.3) after bleaching, indicating that none of the tested materials maintained clinically acceptable color stability following repeated at-home bleaching.

All the materials demonstrated clinically unacceptable color changes; however, the magnitude and rate of ΔE increase varied among materials, allowing for comparison of relative performance under bleaching conditions.

Following the first bleaching cycle, Admira Fusion, Filtek Supreme Ultra, and Omnichroma bonded with Scotchbond Universal Bond exhibited higher early ΔE values compared with Harmonize and Omnichroma bonded with Tokuyama Universal Bond (Table 2). Despite these differences, all initial ΔE values already exceeded clinical acceptability thresholds, suggesting early bleaching-induced color alteration across materials.

By the third bleaching cycle, a range of approximately 5.0 to 8.0 was observed for ΔE values. All the materials’ color changes were well beyond clinically acceptable limits. Comparisons among materials at this stage focused on their relative susceptibility to bleaching rather than their ability to maintain acceptable esthetic performance. After the fifth bleaching cycle, ΔE values converged for most materials. Omnichroma bonded with Scotchbond Universal Bond demonstrated the greatest overall color change (mean ΔE ≈ 11.3), whereas Omnichroma bonded with Tokuyama Universal Bond exhibited comparatively lower ΔE values; however, both remained clinically unacceptable.

A repeated-measures ANOVA was conducted to characterize further time-dependent color changes for each restorative material (Table 3). It demonstrated a statistically significant effect of the bleaching cycle on ΔE for most materials (*p* < 0.001), with moderate to very large effect sizes. Filtek Supreme Ultra exhibited a smaller, statistically non-significant time-dependent effect; however, its ΔE values still exceeded clinical acceptability thresholds, reinforcing that statistical non-significance does not equate to clinical acceptability. (Table 3).

Overall, differences among materials should be interpreted as differences in the extent of bleaching-induced color change rather than as differences in the preservation of clinically acceptable color stability.

### 3.2. Surface Roughness (Ra)

**Figure 3:**
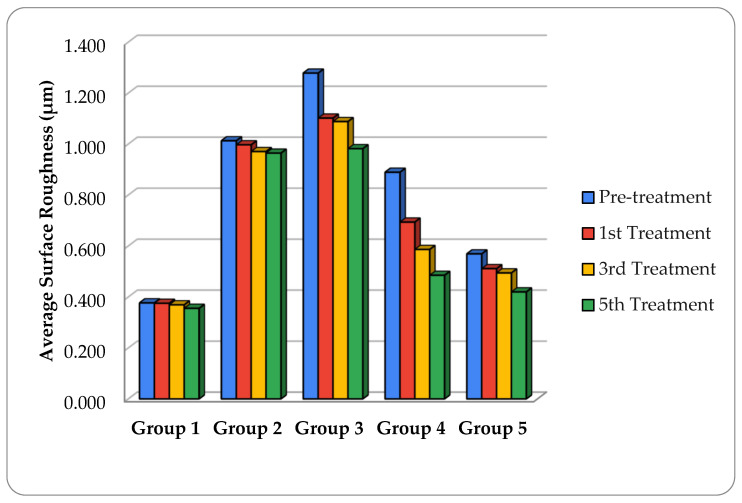
Mean surface roughness (Ra, µm) of restorative materials across bleaching cycles, illustrating relative material-dependent surface changes.

The mean surface roughness (Ra) values before and after bleaching cycles are presented in Figure 3. Ra values generally decreased with repeated bleaching cycles across all groups; however, the magnitude of change varied among materials. The highest Ra values observed were for group 3 (Harmonize) and group 2 (Filtek Supreme Ultra) at baseline. On the other hand, Admira Fusion showed the lowest initial surface roughness. Following bleaching, Ra levels decreased in all materials. Group 1 (Admira Fusion) and Group 5 (Omnichroma + Tokuyama Universal Bond) maintained the lowest Ra values throughout the experimental period and exhibited minimal overall surface alteration.

The repeated measures ANOVA demonstrated a statistically significant effect of the bleaching cycle on Ra for group 2 (Filtek Supreme Ultra: *p* ≤ 0.007), group 3 (Harmonize: *p* ≤ 0.001), and for group 4 (Omnichroma + Scotchbond Universal Bond: *p* ≤ 0.001), with small to moderate effect sizes (ηp^2^ = 0.20–0.29) (Table 4). In contrast, no statistically significant changes in Ra were detected for Admira Fusion (*p* = 0.834; ηp^2^ = 0.01) or Omnichroma bonded with Tokuyama Universal Bond (*p* = 0.217; ηp^2^ = 0.07) (Table 4).

Although statistically significant reductions in Ra were observed for several materials, the absolute magnitude of change was limited; therefore, statistical significance reflects measurable surface modification rather than necessarily clinically meaningful surface degradation or improvement.

### 3.3. Material- and Adhesive-Dependent Trends

Bleaching-induced color changes and surface roughness were material-dependent. The structurally colored single-shade composite group 4 (Omnichroma + Scotchbond) exhibited the greatest susceptibility to bleaching-induced color change, whereas group 5 (Omnichroma + Tokoyama) showed the least. These findings suggest that adhesive selection influences the response of the single-shade composite, with Tokuyama Universal Bond associated with reduced color change and surface roughness alteration compared with Scotchbond Universal Bond, although adhesive-related effects should be interpreted as exploratory due to study design constraints. Despite these differences, all materials exhibited clinically unacceptable color changes following repeated bleaching cycles, and no restorative material or adhesive system completely prevented bleaching-induced esthetic changes.

Therefore, statistically significant differences among materials reflect differences in the magnitude of bleaching-induced changes rather than the preservation of clinically acceptable esthetic properties.

## 4. Discussion

This study evaluated the effects of at-home carbamide peroxide bleaching on color stability and surface roughness of a single-shade resin composite, an ORMOCER-based composite, and a conventional nanohybrid composite.

Statistically significant differences among materials in this study do not constitute evidence of clinical acceptability, particularly because all materials exceeded established color acceptability thresholds. Therefore, differences reflect relative susceptibility rather than preservation of esthetic integrity. Although statistically significant reductions in surface roughness were observed, these changes should not be interpreted as surface improvement, as bleaching-induced resin matrix degradation may produce superficial smoothing without enhancing long-term material durability.

The bleaching resulted in measurable changes in color and surface characteristics across all tested materials. More importantly, all materials exceeded the established clinical acceptability thresholds, particularly for color change after repeated bleaching cycles. Under the experimental conditions of this study, none of the materials maintained clinically acceptable esthetic stability following repeated bleaching cycles. Therefore, differences among materials should be interpreted as susceptibility to bleaching-induced changes rather than as evidence of clinical acceptability.

The development of single-shade composite resin systems was driven by the objective of enhancing clinical efficiency by reducing procedural complexity and chairside time. Recent research examining the effect of aging and bleaching of single -shade composite versus multi-shade composite indicated that bleaching could partially reduce the color stability of the composite resins [18]. These materials reduce shade selection variability by relying on structural color effects, controlled light scattering, and engineered filler particles with refractive indices optimized to interact with the resin matrix and surrounding tooth structure [3,19,20,21,22]. In the present study, the single-shade composite Omnichroma showed color changes following bleaching that were comparable to or exceeded those observed in conventional nanohybrid composites. Omnichroma showed satisfactory initial color adaptation; however, subsequent bleaching cycles led to a gradual increase in ΔE values, ultimately exceeding clinically acceptable limits. These findings align with previous reports suggesting that structurally colored single-shade composites may be more sensitive to changes in their optical environment following bleaching and aging due to alterations in filler–matrix refractive index interactions [23,24]. Even though Omnichroma bonded with Tokuyama Universal Bond showed less color change than the same composite bonded with Scotchbond Universal Bond, the resulting ΔE values were, however, still exceeding the clinically acceptable thresholds. This finding indicates that while adhesive selection may reduce bleaching-related color changes, it does not fully prevent bleaching-induced color instability.

Conventional nanohybrid composites, including Filtek Supreme Ultra and Harmonize, demonstrated some color instability. The Filtek Supreme Ultra exhibited a minor, non-significant time-dependent effect in repeated-measures analysis; its ΔE values still exceeded the established acceptability thresholds. These findings are in agreement with previous studies reporting bleaching-induced color instability in conventional resin composites [19,25,26,27,28]. From a clinical standpoint, at-home bleaching may compromise the esthetic stability of existing composite restorations regardless of material type. None of the examined materials maintained clinically acceptable color stability following repeated bleaching cycles. Clinicians should therefore anticipate the possible need for restoration repolishing, adjustment, or replacement after bleaching, particularly in highly esthetic regions [5,7]. This consideration is particularly important when using single-shade composites in patients who frequently undergo tooth whitening procedures [29].

For the surface roughness, bleaching resulted in either minimal change or a statistically significant reduction in Ra across materials. The observed reduction in surface roughness may be attributed to superficial resin matrix softening, filler–matrix interface degradation, and partial leveling of surface irregularities rather than true surface improvement. Reductions in Ra following bleaching have been reported for both enamel and resin-based materials, and may reflect chemical degradation at the resin–filler interface rather than mechanical surface wear [30,31,32]. Smoother surfaces may be advantageous for reduced plaque accumulation and staining; however, reduced roughness should not be interpreted as improved material durability, as bleaching may still compromise other properties, such as microhardness or marginal integrity. In contrast, the present study found that surface roughness decreased across all tested groups, with Admira Fusion showing the smallest change. This difference may be explained by the finishing and polishing protocol employed, as high-quality finishing reduces surface irregularities and may buffer bleaching-induced degradation [32].

Admira Fusion and Omnichroma bonded with Tokuyama Universal Bond exhibited the least change in surface roughness, suggesting greater relative resistance to bleaching-induced surface modification. The enhanced surface stability of the ORMOCER-based composite may be attributed to its highly cross-linked inorganic–organic network structure, which has been associated with increased resistance to oxidative and hydrolytic degradation [33]. The observation in the present study, that adhesive selection influences surface roughness and color stability, highlights the adhesive interface’s role in esthetic performance of composite restorations under bleaching conditions.

While the present study evaluated color change and surface roughness, other clinically relevant properties, including microhardness, marginal integrity, degree of conversion, thermomechanical ageing behaviour, and long-term colour stability under staining or smoke exposure, were not assessed. Previous studies have shown that bleaching can adversely affect these properties and may contribute to the degradation of restorations over time [11,31,34]. Furthermore, the standardized Class I cavity preparations in the experimental design may have introduced experimental consistency; however, it may also have led to anatomical variability that could have influenced surface mapping and profilometric measurements.

### Limitations

This study has inherent limitations that restrict direct clinical extrapolation. The in vitro design did not simulate thermocycling, mechanical loading, or long-term intraoral ageing. Only one bleaching concentration and protocol was evaluated, and the absence of a non-bleached control group limits the ability to isolate bleaching-specific effects from storage-related changes. The experimental model focused exclusively on bleaching and did not account for common clinical discoloration challenges such as staining beverages or tobacco smoke exposure. The small standardized Class I cavity dimensions and anatomical variability among extracted molars may have influenced optical and surface measurements. Surface roughness assessment was limited to Ra values without complementary microstructural imaging, and other clinically relevant properties, including microhardness, wear resistance, gloss retention, marginal integrity, and patient-centred esthetic outcomes, were not assessed. Adhesive-related findings should be interpreted cautiously, as this study was not powered to independently evaluate the effects of adhesive systems. Future studies incorporating combined ageing models and longer observation periods are needed to better approximate clinical performance.

Overall, the results of this study indicate that bleaching-induced esthetic alteration occurs across restorative materials, and differences among materials should be interpreted as differences in the degree of change rather than as evidence of acceptable esthetic stability. Although it has been demonstrated that certain materials exhibit greater resistance to bleaching-related effects, clinicians should be cautious when planning restorative treatment for patients considering bleaching.

## 5. Conclusions

Within the limitations of this study, all restorative materials exhibited color changes exceeding established clinical acceptability thresholds after repeated at-home bleaching, with the magnitude of change varying by material. Omnichroma bonded with Scotchbond Universal demonstrated the greatest color change, while Tokuyama Universal Bond was associated with comparatively lower ΔE values, although adhesive-related effects should be interpreted as exploratory. ORMOCER-based composites demonstrated greater resistance to surface roughness changes; however, no material or adhesive system prevented bleaching-related esthetic alterations.

## Figures and Tables

**Table 1:** Description of the composites and bonding agents.

Group #	Material/Manufacturer	Matrix	Filler Type/Size	Filler (% wt/vol)	Bonding Agent
*Group 1*	Admira Fusion (VOCO GmbH, Cuxhaven, Germany)	Ormocer^®^ resin	Silicon oxide nanofillers; glass ceramic (~1 µm)	84/69	Scotchbond Universal Bond (3M, USA)
*Group 2*	Filtek Supreme Ultra (3M ESPE, Saint Paul, MN, USA)	Bis-GMA, UDMA, Bis-EMA	Silica & zirconia nanoparticles (~20 µm)	72.5/55.9	Scotchbond Universal Bond (3M, USA)
*Group 3*	Harmonize (Kerr, Corporation, Orange, CA, USA)	Bis-GMA, Bis-EMA, TEGDMA	Silica/zirconia nanoparticles (5–400 nm); barium glass (~400 nm)	81/64.5	OptiBond Universal (Kerr, USA)
*Group 4*	Omnichroma (Tokuyama Dental Corp., Tokyo, Japan)	UDMA, TEGDMA	Supra-nano spherical SiO_2_–ZrO_2_ (~260 nm)	79/68	Scotchbond Universal Bond (3M, USA)
*Group 5*	Omnichroma + Tokuyama Universal Bond (Tokuyama Dental Corp., Japan)	UDMA, TEGDMA	Supra-nano spherical SiO_2_–ZrO_2_ (~260 nm)	79/68	Tokuyama Universal Bond (Tokuyama, Japan)

**Table 2:** Mean color change (ΔE ± SD) across bleaching cycles, with all materials exceeding clinical acceptability thresholds.

Group # (Material)	Cycle 1	Cycle 2	Cycle 3	Cycle 4	Cycle 5
Group 1 (Admira Fusion)	5.8 * ± 0.9	7.1 * ± 1.1	8.0 * ± 1.3	8.4 *± 1.4	9.1 * ± 1.5
Group 2 (Filtek Supreme Ultra)	6.7 * ± 1.0	6.8 * ± 1.0	7.2 * ± 1.2	8.1 * ± 1.3	8.7 * ± 1.4
Group 3 (Harmonize)	4.0 * ± 0.7	5.4 * ± 1.0	7.4 * ± 1.2	8.8 * ± 1.4	9.2 * ± 1.5
Group 4 (Omnichroma + Scotchbond)	6.0 * ± 1.0	7.1 * ± 1.2	7.9 * ± 1.4	10.1 * ± 1.6	11.3 * ± 1.7
Group 5 (Omnichroma + Tokuyama)	3.1 * ± 0.8	4.5 * ± 1.0	5.0 * ± 1.1	6.8 * ± 1.3	7.6 *± 1.4

* (ΔE values > 3.3 were considered clinically unacceptable).

**Table 3:** Repeated-measures ANOVA assessing the effect of bleaching cycle on color change (ΔE).

Group # (Material)	F(df_1_, df_2_)	*p*-Value	ηp^2^	Interpretation
Group 1 (Admira Fusion)	F(3, 102) = 12.61	<0.001 *	0.27	Moderate shade change
Group 2 (Filtek Supreme Ultra)	F(3, 27) = 1.74	0.182	0.16	Statistically non-significant change despite clinically unacceptable ΔE
Group 3 (Harmonize)	F(3, 27) = 14.13	<0.001 *	0.41	Strong shade changes
Group 4 (Omnichroma + Scotchbond)	F(3, 102) = 50.50	<0.001 *	0.72	**Very strong shade change**
Group 5 (Omnichroma + Tokuyama)	F(3, 42) = 16.41	<0.001 *	0.54	**Strong shade change**, improved retention compared to group 4

* *p* > 0.05; ηp^2^ is partial eta squared, standard effect size.

**Table 4:** Repeated-measures ANOVA assessing the effect of bleaching cycle on surface roughness (Ra).

Group # (Material)	F(df_1_, df_2_)	*p*-Value	ηp^2^	Interpretation
Group 1 (Admira Fusion)	F(3, 102) = 0.29	0.834	0.01	No significant change across cycles
Group 2 (Filtek Supreme Ultra)	F(3, 27) = 4.96	<0.007 *	0.20	Decrease across cycles
Group 3 (Harmonize)	F(3, 27) = 7.66	<0.001 *	0.28	Decrease across cycles
Group 4 (Omnichroma + Scotchbond)	F(3, 102) = 8.19	<0.001 *	0.29	Decrease across cycles
Group 5 (Omnichroma + Tokuyama)	F(3, 42) = 1.54	0.217	0.07	No significant change

* *p* > 0.05; ηp^2^ is partial eta squared, standard effect size.

## Data Availability

The original contributions presented in this study are included in the article. Further inquiries can be directed to the corresponding author.
